# Development and validation of nomograms for predicting survival in patients with non-metastatic colorectal cancer

**DOI:** 10.18632/oncotarget.16167

**Published:** 2017-03-13

**Authors:** Huihong Jiang, Erjiang Tang, Dan Xu, Ying Chen, Yong Zhang, Min Tang, Yihua Xiao, Zhiyong Zhang, Xiaxing Deng, Huaguang Li, Moubin Lin

**Affiliations:** ^1^ Department of General Surgery, Ruijin Hospital Affiliated to Shanghai Jiaotong University School of Medicine, Shanghai, China; ^2^ Center for Translational Medicine, Yangpu Hospital, Tongji University School of Medicine, Shanghai, China; ^3^ Department of General Surgery, Yangpu Hospital, Tongji University School of Medicine, Shanghai, China; ^4^ Department of General Surgery, Zhuji People's Hospital of Zhejiang Province, Zhejiang, China

**Keywords:** colorectal cancer, nomogram, prognostic, overall survival, cancer-specific survival

## Abstract

**Background:**

This study aimed to develop nomograms for predicting survival in patients with non-metastatic colorectal cancer (CRC).

**Results:**

On multivariate analyses of the derivation set, the nomograms for OS and CSS shared common significant prognostic factors: age, first-degree relative cancer history, differentiation grade, vessels/nerves invasion, TNM stage, CEA, CA19-9 and PNI. The nomograms displayed good accuracy in predicting OS and CSS, with C-indexes of 0.75 and 0.76, respectively. The calibration plots also showed an excellent agreement between the predicted and observed survival probabilities. Furthermore, the predictive accuracy of the nomograms was confirmed in the validation set, with C-indexes of 0.79 and 0.83 for OS and CSS, respectively.

**Materials and Methods:**

On the basis of data from 822 patients with resected non-metastatic CRC, nomograms for predicting overall survival (OS) and cancer-specific survival (CSS) were established using Cox regression model. The predictive performance of the nomograms was assessed by concordance index (C-index) and calibration plot. An independent external cohort of 171 patients was used to validate the nomograms.

**Conclusions:**

We developed and validated two nomograms for patients with non-metastatic CRC, which could provide individual prediction of OS and CSS with high accuracy.

## INTRODUCTION

Colorectal cancer (CRC) is the third most frequently diagnosed cancer in males and the second in females, with an estimated 1.4 million new cases and 693,900 deaths worldwide in 2012 [1]. Although some advances have been made in the treatment of CRC over the decades [2, 3], local recurrence and distant metastases continue to be a formidable challenge for clinicians [4]. Tumor-node-metastasis (TNM) staging system is the most basic and prevalent for predicting prognosis of CRC patients undergoing radical surgery, whereas the predictive accuracy is limited, particularly in patients with localized disease [5]. It has been gradually recognized that some other clinical factors could significantly contribute to individual prediction of prognosis, such as age, histology, systemic inflammation and nutritional status [6, 7].

Nomograms have been accepted as reliable and pragmatic prediction tools to quantify individual risk by incorporating multiple significant prognostic factors, and have been shown to achieve good predictive performance in a variety of cancers, such as hepatocellular carcinoma [8], lung cancer [9], breast cancer [10] and gastric cancer [11]. However, nomograms for predicting survival in patients with localized CRC have been relatively few to date [12]. In this study, we aimed to identify readily available clinical factors most helpful in predicting survival of patients with non-metastatic CRC, and to develop prognostic nomograms that can serve as a useful guide in patient management.

## RESULTS

### Patient characteristics

The demographic and clinical characteristics of the derivation and validation patient cohorts were summarized in Supplementary Table 1. Of the 822 patients in the derivation set, 477 (58.0%) were males and 345 (42.0%) were females, with a median age of 63 years (range 22–92). There were 177 (21.5%) patients with stage I disease, 322 (39.2%) patients with stage II disease, and 323 (39.3%) patients with stage III disease. During the median follow-up of 68.5 months, there were 240 (29.2%) deaths and 189 (23.0%) cancer-related deaths. Among the 171 patients in the validation set, 42 (23.6%) were in stage I, 68 (39.8%) were in stage II, and 61 (35.7%) were in stage III. The median follow-up time was 69.5 months, and 56 (31.0%) patients had died at the last follow-up, of which 46 (26.9%) deaths were cancer-related. Patient characteristics in the two cohorts were well balanced.

### Development of the nomogram

By univariate analyses, ten of eighteen clinical variables were found to be associated with overall survival (OS) and cancer-specific survival (CSS) (*P* < 0.05), and were advanced forward (Table 1). The multivariate Cox regression model with a stepwise selection procedure identified that the following eight of ten prognostic factors were the strongest independent predictors for both OS and CSS: age, first-degree relative cancer history, differentiation grade, vessels/nerves invasion, TNM stage, carcinoembryonic antigen (CEA), carbohydrate antigen (CA) 19–9 and Onodera's prognostic nutritional index (PNI) (Table 2).

**Table 1:** Univariate analyses for OS and CSS in the derivation set

Variable	OS		CSS	
	HR (95% CI)	*P* value	HR (95% CI)	*P* value
Age				
< 65	1.00		1.00	
65–74	1.55 (1.13–2.12)	0.007	1.49 (1.05–2.12)	0.025
≥ 75	2.29 (1.72–3.05)	< 0.001	2.00 (1.44–2.78)	< 0.001
Sex				
Female vs. Male	0.86 (0.67–1.10)	0.233	0.91 (0.69–1.21)	0.538
Smoking history				
Yes vs. No	0.95 (0.69–1.30)	0.740	1.04 (0.74–1.47)	0.818
Alcohol-drinking history				
Yes vs. No	0.86 (0.61–1.20)	0.376	0.92 (0.63–1.32)	0.639
First-degree relative cancer history				
Yes vs. No	0.59 (0.38–0.92)	0.019	0.69 (0.43–0.94)	0.031
Tumor site				
Colon vs. Rectum	1.19 (0.93–1.53)	0.160	1.21 (0.92–1.61)	0.171
Differentiation grade				
Poor/mucinous vs. Well/moderate	1.97 (1.52–2.54)	< 0.001	2.43 (1.83–3.22)	< 0.001
Vessels/nerves invasion				
Positive vs. Negative	2.48 (1.78–3.44)	<0.001	2.73 (1.90–3.92)	< 0.001
TNM stage				
I	1.00		1.00	
II	2.10 (1.30–3.41)	0.003	1.98 (1.14–3.46)	0.016
III	4.53 (2.87–7.14)	< 0.001	4.99 (2.97–8.40)	< 0.001
CEA				
> 5.0 vs. ≤ 5.0	2.01 (1.57–2.57)	< 0.001	2.40 (1.81–3.19)	< 0.001
CA19-9				
> 37.0 vs. ≤ 37.0	2.44 (1.85–3.20)	< 0.001	2.78 (2.06–3.76)	< 0.001
WBC				
≥ 4.0 vs. < 4.0	1.02 (0.62–1.66)	0.950	1.04 (0.60–1.83)	0.878
HGB				
≥ 120.0 vs. < 120.0	0.66 (0.52–0.85)	0.001	0.62 (0.47–0.82)	< 0.001
PLR				
< 114	1.00		1.00	
114–193	1.39 (1.03–1.88)	0.031	1.55 (1.09–2.20)	0.014
> 193	1.96 (1.43–2.70)	< 0.001	2.36 (1.64–3.39)	0.012
PNI				
< 37	1.00		1.00	
37–45	0.45 (0.32–0.61)	< 0.001	0.53 (0.36–0.78)	0.001
> 45	0.26 (0.18–0.37)	< 0.001	0.30 (0.20–0.45)	< 0.001
TB				
> 17.1 vs. ≤ 17.1	0.82 (0.64–1.06)	0.130	0.78 (0.59–1.05)	0.101
ALT				
> 40 vs. ≤ 40	0.93 (0.52–1.66)	0.812	0.99 (0.52–1.87)	0.971
AST				
> 40 vs. ≤ 40	1.37 (0.73–2.57)	0.333	1.40 (0.69–2.84)	0.351

Abbreviations: OS, overall survival; CSS, cancer-specific survival; HR, hazard ratio; CI, confidence interval; vs, versus; TNM, tumor-node-metastasis; CEA, carcinoembryonic antigen; CA19-9, carbohydrate antigen 19-9; WBC, white blood cell; HGB, hemoglobin; PLR, Platelet-to-lymphocyte ratio; PNI, Onodera's prognostic nutritional index; TB, total bilirubin; ALT, alanine aminotransferase; AST, aspartate aminotransferase.

**Table 2:** Multivariate analyses for OS and CSS in the derivation set

Variable	OS		CSS	
	HR (95% CI)	*P* value	HR (95% CI)	*P* value
Age				
< 65	1.00		1.00	
65–74	1.52 (1.11–2.09)	0.010	1.49 (1.04–2.12)	0.029
≥ 75	2.17 (1.61–2.92)	< 0.001	1.96 (1.40–2.76)	< 0.001
First-degree relative cancer history				
Yes vs. No	0.61 (0.39–0.95)	0.030	0.67 (0.42–0.97)	0.041
Differentiation grade				
Poor/mucinous vs. Well/moderate	1.62 (1.25–2.10)	< 0.001	1.96 (1.47–2.61)	< 0.001
Vessels/nerves invasion				
Positive vs. Negative	1.92 (1.37–2.69)	< 0.001	2.05 (1.41–2.98)	< 0.001
TNM stage				
I	1.00		1.00	
II	1.63 (1.06–2.67)	0.038	1.39 (1.02–2.46)	0.044
III	3.30 (2.05–5.29)	< 0.001	3.16 (1.84–5.44)	< 0.001
CEA				
> 5.0 vs. ≤ 5.0	1.36 (1.04–1.78)	0.024	1.60 (1.18–2.18)	0.002
CA19–9				
> 37.0 vs. ≤ 37.0	1.59 (1.19–2.13)	0.002	1.74 (1.26–2.41)	< 0.001
PNI				
< 37	1.00		1.00	
37–45	0.55 (0.39–0.76)	< 0.001	0.64 (0.43–0.95)	0.025
> 45	0.40 (0.28–0.57)	< 0.001	0.45 (0.30–0.69)	< 0.001

Abbreviations: OS, overall survival; CSS, cancer-specific survival; HR, hazard ratio; CI, confidence interval; vs, versus; TNM, tumor-node-metastasis; CEA, carcinoembryonic antigen; CA19-9, carbohydrate antigen 19-9; PNI, Onodera's prognostic nutritional index.

**Figure 1:**
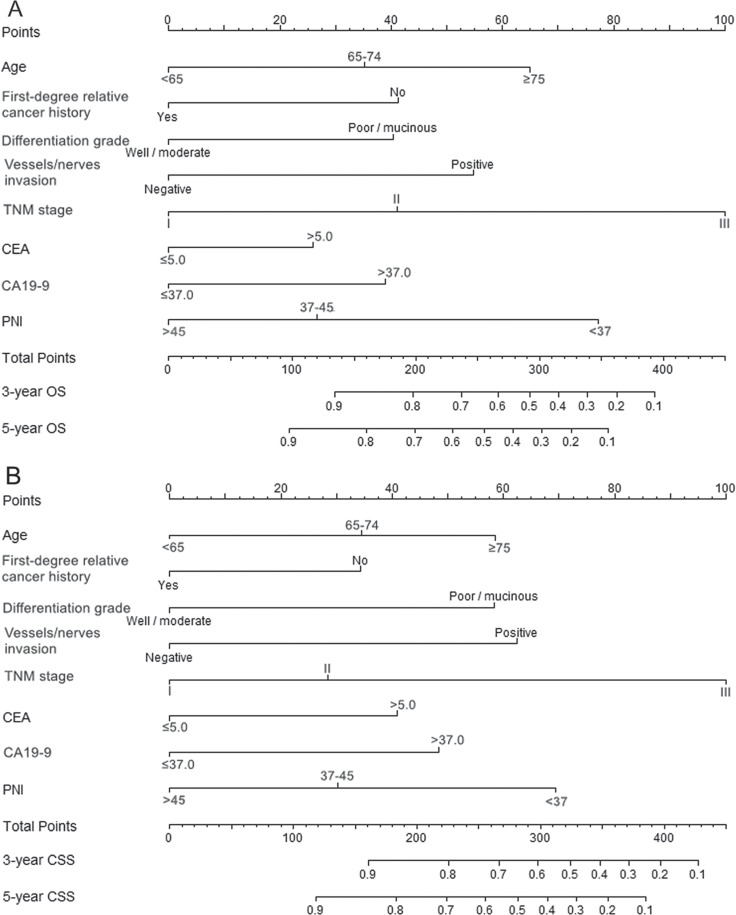
Nomograms for predicting OS (**A**) and CSS (**B**) based on the derivation set. The nomogram is used by adding up the points identified on the points scale for each variable. According to the sum of these points projected on the bottom scales, the nomogram can provide the probabilities of 3- and 5-year OS and CSS for an individual patient.

**Figure 2:**
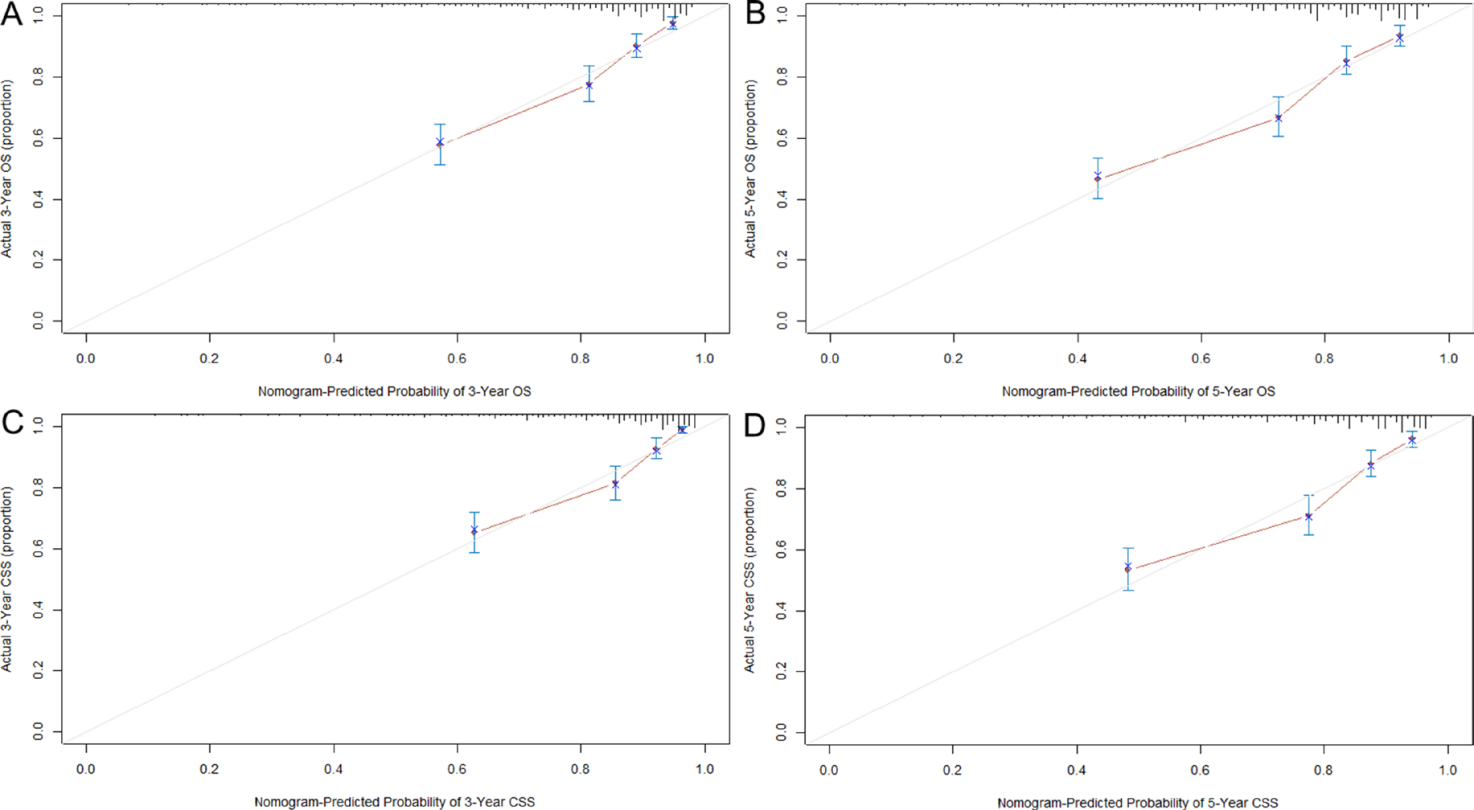
Calibration curves for predicting OS (**A** and **B**) and CSS (**C** and **D**) at 3 and 5 years in the derivation set. The 45-degree straight line represents the perfect match between the actual (Y-axis) and nomogram-predicted (X-axis) survival probabilities. A closer distance between two curves indicates higher accuracy.

Prognostic nomograms were then developed based on the eight significant predictors (Figure 1). In the derivation set, the nomogram displayed good accuracy in the prediction of OS and CSS, with concordance indexes (C-indexes) of 0.75 (95% CI 0.72–0.78) and 0.76 (95% CI 0.73–0.80), respectively. The calibration plots also presented an excellent agreement between the nomogram prediction and actual observation in the probabilities of 3- and 5-year OS and CSS (Figure 2).

### Validation of the nomogram

**Figure 3:**
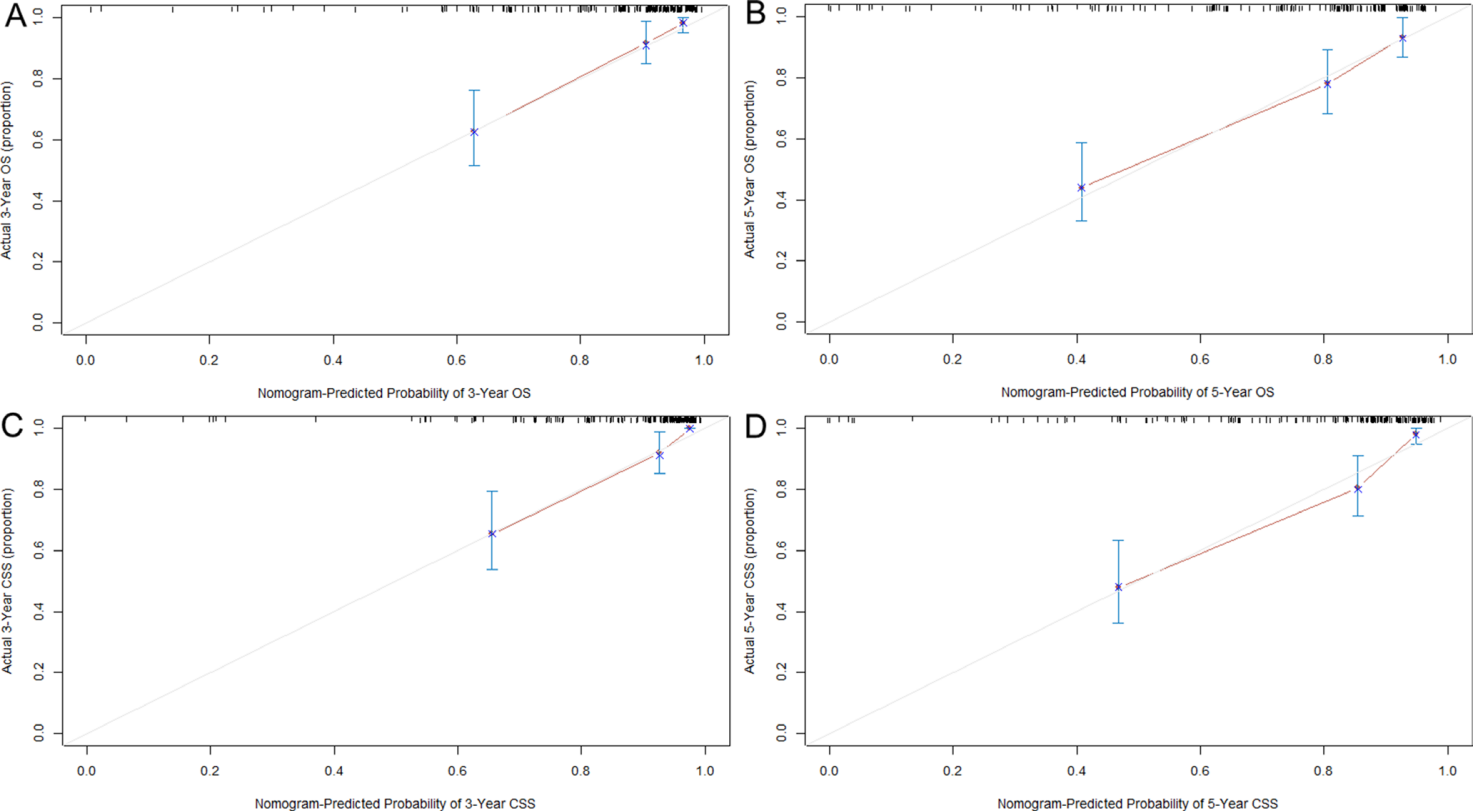
Calibration curves for predicting OS (**A** and **B**) and CSS (**C** and **D**) at 3 and 5 years in the validation set. The 45-degree straight line represents the perfect match between the actual (Y-axis) and nomogram-predicted (X-axis) survival probabilities. A closer distance between two curves indicates higher accuracy.

To further evaluate the predictive power, the nomograms were applied to an independent validation cohort. The C-indexes of the nomograms for predicting OS and CSS reached 0.79 (95% CI 0.74–0.85) and 0.83 (95% CI 0.78–0.88), respectively. Furthermore, the calibration plots showed that the predicted probabilities of OS and CSS at 3 and 5 years agreed well with the actual observations (Figure 3).

## DISCUSSION

In the present study, we constructed nomograms to predict OS and CSS for patients with non-metastatic CRC receiving curative resection. The nomograms consistently achieved considerable predictive accuracy and appreciable reliability in both derivation and validation sets.

Due to the high incidence and risk of recurrence or metastasis, CRC remains a substantial public health burden worldwide [1, 13]. Improved strategies to identify patients at high risk of poor survival are urgently needed. Here, we developed two nomograms that defined a meaningful range of prognostic factors, which were all rapidly available in daily clinical practice. Moreover, to avoid overfitting, a combination of Cox regression model and AIC was used to identify factors that contributed most to the prognostic nomograms [14]. Eight factors were finally incorporated into our nomograms, including age, first-degree relative cancer history, differentiation grade, vessels/nerves invasion, TNM stage, CEA, CA19-9 and PNI. They have all been previously reported to be significantly associated with cancer prognosis [6, 15–17], but our study is the first to incorporate them together for modeling. Of note, PNI, which reflects the immune and nutritional status of host, has an important weight in the nomograms. This result is consistent with previous finding that systemic inflammation and nutritional status play important roles in the prognosis of CRC patients [7, 18, 19].

Nomograms provide a simple and graphic representation of complicated statistical model to quantify individual risk, and show a wide application prospect in clinical practice and research [20]. Although several nomograms have been constructed to predict survival for patients with localized CRC, the predictive accuracy is not entirely satisfactory. Valentini *et al*. [21] established a nomogram derived from analysis of 2242 patients with locally advanced rectal cancer, which included age, sex, clinical T stage, pathologic T stage, pathologic N stage, adjuvant chemotherapy, surgery procedure and radiotherapy dose. However, the nomogram showed moderate accuracy in OS prediction, with C-indexes of 0.68 and 0.70 in the training and validation sets, respectively. Peng *et al*. [22] also developed a nomogram to predict OS for patients with locally advanced rectal cancer, but the C-index only reached 0.70. Factors of the nomogram included age, gender, CEA, tumor location, T stage, N stage, ratio of metastatic lymph nodes, adjuvant chemotherapy and adjuvant chemoradiotherapy. In the current study, we established two nomograms for patients with non-metastatic colorectal cancer (CRC), which displayed good accuracy with a C-index of 0.75 for OS and 0.76 for CSS. Furthermore, the excellent predictive performance in an external validation set guaranteed the repeatability and reliability of the nomograms. Our nomograms would allow clinicians to identify patients at high risk of poor survival before the treatment, and to make better clinical decisions and follow-up surveillance for patients.

Some limitations of our study should be mentioned. First, because of the retrospective design of the study, potential selection bias is unavoidable. Second, other prognostic factors not included in this study cannot be examined for confounding, which may place a limitation on the survival analysis. Last but not least, the sample size of the validation set is a little small, and it may affect the credibility of assessment results to some extent. Hence, further efforts on prospective data collection and incorporation of more well-recognized predictors are encouraged to improve the model performance.

In conclusion, we developed and externally validated two prognostic nomograms for patients with non-metastatic CRC, which could provide individual prediction of OS and CSS with high accuracy.

## MATERIALS AND METHODS

### Study population

This study included a total of 993 patients with histologically confirmed, non-metastatic CRC (stage I–III). Of these, 822 patients were enrolled from Ruijin Hospital affiliated to Shanghai Jiaotong University School of Medicine between January 2008 and December 2010 and were used to form the basis for the modeling, and 171 patients recruited from Zhuji People's Hospital of Zhejiang Province from January 2007 to December 2010 were used as a validation set. All patients were newly diagnosed and underwent radical surgery, with follow-up to January 2016. To minimize heterogeneity in the study population, patients were excluded from the study if they had received previous anticancer treatment, or had another malignancy, end-stage liver disease or chronic inflammatory disease including autoimmune disorder and infection.

### Data collection

A series of demographic and clinical characteristics were abstracted from patients’ medical records, including age, sex, smoking history, drinking history, family history of cancer, date of diagnosis, tumor site, differentiation grade, vessels/nerves invasion and tumor stage. Tumor was staged according to the American Joint Committee on Cancer (AJCC) TNM classification (Version 7.0). Ten selected preoperative laboratory indexes that may be associated with cancer prognosis were also recorded as follow: CEA, CA 19-9, white blood cell (WBC), lymphocyte, platelet, hemoglobin (HGB), albumin, total bilirubin (TB), alanine aminotransferase (ALT) and aspartate aminotransferase (AST). Platelet-to-lymphocyte ratio (PLR) [23] and PNI [15] were calculated as platelet count (per mL) / lymphocyte count (per mL) and 10 × albumin (g/dL) + 0.005 × lymphocyte count (per mL), respectively. Information on vital status was obtained from the medical records or telephone follow-up. This study was conducted according to the principles of Declaration of Helsinki [24] and was approved by the Research Ethics Committees of both Ruijin Hospital and Zhuji Hospital. All participants provided written informed consent.

### Statistical analysis

The study endpoints included OS and CSS. OS was defined as the interval from diagnosis to death, regardless of the cause. CSS was calculated from the date of diagnosis to death from cancer or complications of treatment. Continuous variables were transformed into categorical variables based on the limits of clinical normality (for CEA, CA 19-9, WBC, HGB, TB, ALT, and AST) or cut-off values derived from X-tile software (Version 3.6.1, Yale University, USA) [25] which determined the optimal categorizations (for age, PLR, and PNI) (Supplementary Figure 1). Statistical analyses were performed using R Version 3.2.0 (http://www.r-project.org/). Chi-squared or Fisher's exact test was used to compare differences in patient characteristics. To identify the significant prognostic factors, Cox proportional hazards model was used to estimate hazard ratios (HRs) and their 95% confidence intervals (CIs) in the derivation set. Nomograms for predicting OS and CSS were then formulated based on the results of multivariate analyses and by using the package of rms in R [26]. The final model selection was performed using Akaike's information criterion (AIC) in a backward stepwise procedure [27]. The predictive performance of the nomograms was measured by C-index [28] and was assessed by comparing nomogram predicted versus (vs) observed probability of survival (illustrated with a calibration curve). Bootstraps with 1000 resamples were applied to these activities. A larger C-index indicated more accurate prognostic stratification. Furthermore, the prognostic nomograms were validated in an independent external cohort. All *P* values were two-sided and values less than 0.05 were judged statistically significant.

## SUPPLEMENTARY MATERIALS FIGURES AND TABLES


