# Correction: Development and validation of nomograms for predicting survival in patients with non-metastatic colorectal cancer

**DOI:** 10.18632/oncotarget.26274

**Published:** 2018-10-19

**Authors:** Huihong Jiang, Erjiang Tang, Dan Xu, Ying Chen, Yong Zhang, Min Tang, Yihua Xiao, Zhiyong Zhang, Xiaxing Deng, Huaguang Li, Moubin Lin

**Affiliations:** ^1^ Department of General Surgery, Ruijin Hospital Affiliated to Shanghai Jiaotong University School of Medicine, Shanghai, China; ^2^ Center for Translational Medicine, Ya ngpu Hospital, Tongji University School of Medicine, Shanghai, China; ^3^ Department of General Surgery, Yangpu Hospital, Tongji University School of Medicine, Shanghai, China; ^4^ Department of General Surgery, Zhuji People's Hospital of Zhejiang Province, Zhejiang, China

**This article has been corrected:** The correct Grant support information is given below:

**GRANT SUPPORT**

This work was supported by grants from the National Natural Science Foundation of China (grant no. 81272480) and the Natural Science Foundation of Shanghai (grant no. 15411969900, no. ZK2015A32, and no. 201540132). This work was also supported by the grant from Shanghai Municipal Commission of Health and Family Planning (201540132).

Original article: Oncotarget. 2017; 8:29857-29864. https://doi.org/10.18632/oncotarget.16167

