# Supplementary material for: Development and validation of nomograms for predicting survival in patients with non-metastatic colorectal cancer
Source: Oncotarget. 2017 Mar 13;8(18):29857–64. doi: 10.18632/oncotarget.16167 (PMC5444709; doi:10.18632/oncotarget.16167)
Supplement: Supplementary file 1 [file oncotarget-08-29857-s001.pdf]

Development and validation of nomograms for predicting survival in patients with non-metastatic colorectal cancer

Supplementary Materials

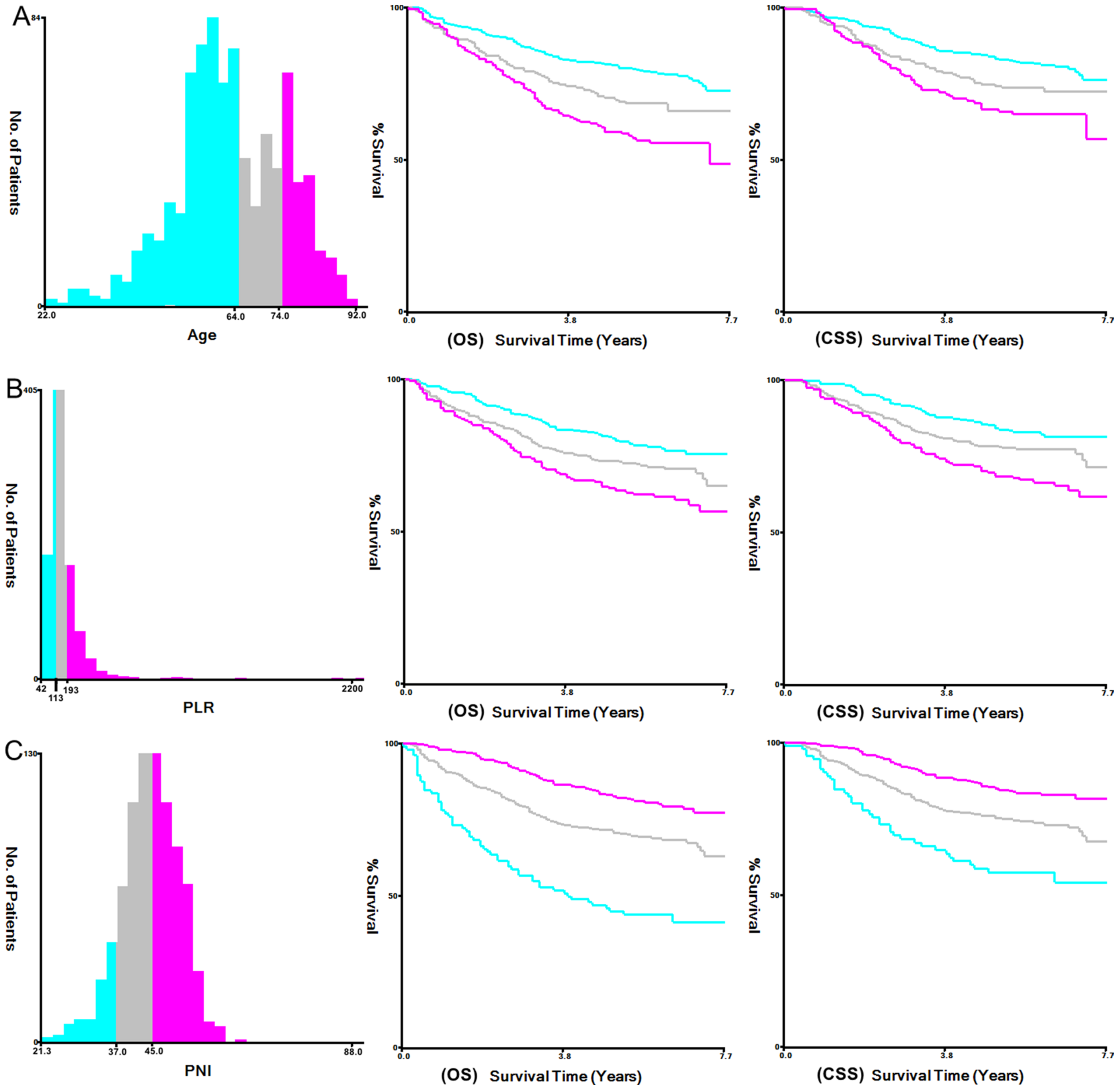

Supplementary Figure 1: X-tile analyses for OS and CSS, according to age (**A**), PLR (**B**), and PNI (**C**). Patients were divided into three distinct prognostic groups based on the optimal cut-off values derived from X-tile analyses (age: < 65, 65–74, ≥ 75; PLR: < 114, 114–193, > 193; PNI: < 37, 37–45, > 45).

Supplementary Table 1: Selected characteristics of patients with non-metastatic CRC

Characteristic	Derivation set (*n* = 822)<br>No. of patients (%)	Validation set (*n* = 171)<br>No. of patients (%)
Age, years		
Median, range	63 (22–92)	64 (33–85)
Sex		
Female	345 (42.0%)	64 (37.4%)
Male	477 (58.0%)	107 (62.6%)
Smoking history		
Yes	157 (19.1%)	39 (22.8%)
No	665 (80.9%)	132 (77.2%)
Alcohol-drinking history		
Yes	150 (18.2%)	38 (22.2%)
No	672 (81.8%)	133 (77.8%)
First-degree relative cancer history		
Yes	110 (13.4%)	22 (12.9%)
No	712 (86.6%)	149 (87.1%)
Tumor site		
Colon	403 (49.0%)	52 (30.4%)
Rectum	419 (51.0%)	119 (69.6%)
Differentiation grade		
Poor/mucinous	212 (25.8%)	26 (15.25)
Well/moderate	610 (74.2%)	145 (84.8%)
Vessels/nerves invasion		
Positive	83 (10.1%)	19 (11.1%)
Negative	739 (89.9%)	152 (88.9%)
TNM stage		
I	177 (21.5%)	42 (24.5%)
II	322 (39.2%)	68 (39.8%)
III	323 (39.3%)	61 (35.7%)
CEA, µg/L		
Median, range	4.1 (0.5–367.1)	4.8 (0.1–146.1)
CA19–9, U/mL		
Median, range	13.8 (0.5–1448.0)	16.5 (0.5–453.6)
WBC, 10^9/L		
Median, range	6.3 (2.3–9.8)	5.9 (2.0–9.4)
HGB, g/L		
Median, range	126 (42–193)	124 (53–164)
PLR		
Median, range	133 (42–2200)	140 (58–514)
PNI		
Median, range	44.5 (21.3–88.0)	48.3 (30.1–60.8)
TB, µmol/L		
Median, range	16 (2.9–111.2)	12.3 (2.6–55.4)
ALT, U/L		
Median, range	16 (5–187)	14 (1–103)
AST, U/L		
Median, range	19 (6–107)	20 (8–82)

Abbreviations: CRC, colorectal cancer; TNM, tumor-node-metastasis; CEA, carcinoembryonic antigen; CA19-9, carbohydrate antigen 19-9; WBC, white blood cell; HGB, hemoglobin; PLR, platelet-to-lymphocyte ratio; PNI, Onodera's prognostic nutritional index; TB, total bilirubin; ALT, alanine aminotransferase; AST, aspartate aminotransferase.
